# Melatonin Abrogates the Anti-Developmental Effect of the AKT Inhibitor SH6 in Bovine Oocytes and Embryos

**DOI:** 10.3390/ijms20122956

**Published:** 2019-06-17

**Authors:** Marwa El Sheikh, Ayman Mesalam, Ahmed Atef Mesalam, Muhammad Idrees, Kyeong-Lim Lee, Il-Keun Kong

**Affiliations:** 1 Department of Animal Science, Division of Applied Life Science (BK21 Plus), Gyeongsang National University, Jinju 52828, Korea; marwa.el-sheikh@hotmail.com (M.E.S.); ahmedatefmesalam@hotmail.com (A.A.M.); idrees1600@gmail.com (M.I.); 2 Department of Microbial Biotechnology, Genetic Engineering and Biotechnology Division, National Research Centre, Dokki, Cairo 12622, Egypt; 3 Department of Theriogenology, Faculty of Veterinary Medicine, Zagazig University, Zagazig 44519, Egypt; aymanmesalam@gmail.com; 4 The King Kong Corp Ltd., Jinju 52828, Korea; 0920-0728@hanmail.net; 5 Institute of Agriculture and Life Science, Gyeongsang National University, Jinju 52828, Korea

**Keywords:** melatonin, AKT, SH6, oocyte, in vitro maturation, bovine, embryo

## Abstract

Melatonin, a nighttime-secreted antioxidant hormone produced by the pineal gland, and AKT, a serine/threonine-specific protein kinase, have been identified as regulators for several cellular processes essential for reproduction. The current study aimed to investigate the potential interplay between melatonin and AKT in bovine oocytes in the context of embryo development. Results showed that the inclusion of SH6, a specific AKT inhibitor, during in vitro maturation (IVM) significantly reduced oocyte maturation, cumulus cell expansion, cleavage, and blastocyst development that were rescued upon addition of melatonin. Oocytes treated with SH6 in the presence of melatonin showed lower levels of reactive oxygen species (ROS) and blastocysts developed exhibited low apoptosis while the mitochondrial profile was significantly improved compared to the SH6-treated group. The RT-qPCR results showed up-regulation of the mRNA of maturation-, mitochondrial-, and cumulus expansion-related genes including GDF-9, BMP-15, MARF1, ATPase, ATP5F1E, POLG2, HAS2, TNFAIP6, and PTGS2 and down-regulation of Bcl-2 associated X apoptosis regulator (BAX), caspase 3, and p21 involved in apoptosis and cell cycle arrest in melatonin-SH6 co-treated group compared to SH6 sole treatment. The immunofluorescence showed high levels of caspase 3 and caspase 9, and low AKT phosphorylation in the SH6-treated group compared to the control and melatonin-SH6 co-treatment. Taken together, our results showed the importance of both melatonin and AKT for overall embryonic developmental processes and, for the first time, we report that melatonin could neutralize the deleterious consequences of AKT inhibition, suggesting a potential role in regulation of AKT signaling in bovine oocytes.

## 1. Introduction

Mammalian oocytes are naturally arrested in their follicles in a quiescent state known as the first meiotic prophase stage, the so-called germinal vesicle (GV) stage, whereas oocyte meiotic maturation refers to the process of transfer from the GV stage to metaphase II (MII) stage. The environment surrounding the oocytes, where the maturation and growth occur, plays an important role in the subsequent developmental processes including chromosomal condensation, cumulus cell expansion and progression from the first meiosis metaphase I (MI) to the second meiosis MII stage followed by arrest at MII stage until fertilization [1,2]. These cellular processes are triggered by signaling cascades involving the action of protein kinases, which are responsible for successful maturation through meiotic resumption of the arrested GV stage oocytes [3,4].

The AKT, also known as protein kinase B (PKB), has been identified as a serine/threonine-specific protein kinase functioning downstream of the activated phosphatidylinositol 3-kinase (PI3K) [5]. The conversion of phosphatidylinositol 4,5-biphosphate PIP2 to phosphatidylinositol 3,4,5-triphosphate PIP3 is mediated by PI3K leading to the formation of phosphoinositide dependent kinase 1 (PDK1). The PDK1 directly mediates the phosphorylation of AKT at Thr308 phosphorylation site or indirectly at Ser473 phosphorylation site by activating mammalian target of rapamycin (mTOR) complex 2, which itself can phosphorylate AKT at Ser473 site [6]. The AKT is considered fully functional when phosphorylated at these two sites [6,7]. In mammalian cells, three closely related isoforms for AKT (AKT1 (PKB-α), AKT2 (PKB-β), and AKT3 (PKB-γ)) have been identified [8,9,10]. Combined AKT knockout mice developed multiple severe growth abnormalities indicating the pivotal role of AKT in cell survival and differentiation [10]. In oocytes, during maturation process, the expression of AKT has been reported in different species including mouse [11,12], bovine [13] and swine [14]. AKT is responsible for the completion of meiosis since it mainly stimulates the transition from MI to MII by activation of cyclin-dependent kinase 1 (CDK1). Inhibition of AKT resulted in meiotic arrest at MI stage in bovine oocytes [13] and hindering the development of embryos [15,16,17] reflecting the vital role of AKT in oocyte maturation. In addition, AKT prevents apoptosis by counteracting p53 and inhibiting the phosphorylation of various pro-apoptotic mediators such as forkhead transcription factors (FOXO), Bcl-2 associated death promotor (BAD) and caspase 9 [18,19,20]. Notably, class I cumulus oocyte complexes (COCs), with five or more layers of cumulus cells showed more AKT activity during early in vitro maturation (IVM) step than class II COCs, with one or two layers of cumulus cells, whereas inhibition of the PI3K/AKT signaling in class I COCs significantly decreased the developmental competence of embryos [21] via reducing oocyte maturation and initiation of apoptosis [17,22,23].

Melatonin (N-acetyl-5-methoxytryptamine) is a hormone originally synthesized and secreted mainly from the pineal gland of vertebrates. It exhibits immunomodulatory and cytoprotective effects as well as being an anti-apoptotic agent [24,25,26,27,28]. As a potent antioxidant, melatonin protects against the oxidative stress of reactive oxygen species (ROS), which is one of the main causes of defective gametes or poorly developed embryos in assisted reproductive technology (ART) [29,30]. The higher oxygen concentration under the in vitro maturation environment leads to production of high concentrations of ROS which can have detrimental effects on developing embryos [31]. Moreover, the antioxidant activity of melatonin improved the quality of the inferior oocytes [32] in addition to the protective role against certain cytotoxic compounds during IVM process [33,34,35,36]. Also, melatonin alleviated the meiotic defects in fetal mouse oocytes [33] and reduced the apoptosis and oxidative stress in bovine ovarian granulosa cells [37]. Though, the possible mechanisms regarding how melatonin improves and rescues the quality and the developmental potential of oocytes have not been fully defined.

Previously, it has been reported that supplementation with certain hormones and antioxidants during IVM affects the developmental competence of bovine embryos via modulation of the PI3K/AKT signaling pathway [17,38,39,40]. The effect of melatonin on the activation of PI3K/AKT signaling pathway was reported in mouse brain astrocytes [41]. Recently, Wang et al. reported the involvement of melatonin in PI3K/AKT pathway in bovine theca cells [42]. Since the possible link between melatonin and AKT signaling in bovine oocytes is still unknown, we first tested the effect of melatonin supplementation and AKT inhibition, using the AKT inhibitor SH6, during in vitro maturation step on the development of bovine embryos. The effect of melatonin supplementation on AKT signaling and developmental competence of SH6-treated oocytes was also investigated.

## 2. Results

### 2.1. Effect of Melatonin and/or SH6 on the Development of pre-Implantation Bovine Embryos In Vitro

**Figure 1:**
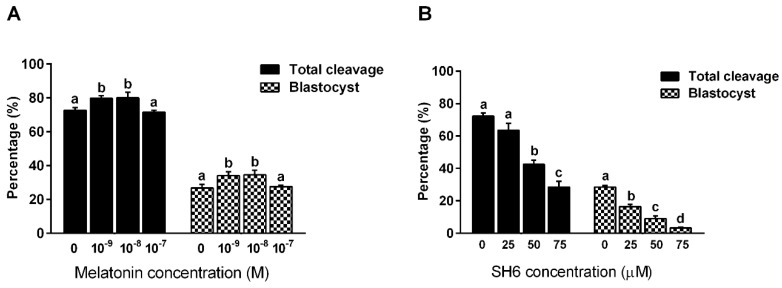
The effect of melatonin and SH6 on the developmental competence of bovine oocytes. Melatonin was added to the maturation medium at concentrations ranging from 10^−9^, 10^−8^ and 10^−7^ M whereas SH6 was used at 25, 50 and 75 µM. (**A**) Total cleavage and day-8 blastocyst rates for serial dilution melatonin experiments; (**B**) Total cleavage and day-8 blastocyst for serial dilution SH6 experiments. Data are expressed as mean ± standard error of the mean (SEM). Values with different superscripts indicate statistically significant difference (*p* < 0.05).

Initially, to elucidate the actual role of melatonin on the developmental competence of bovine embryos, serial dilutions of melatonin (0, 10^−9^, 10^−8^, 10^−7^ M) were added to IVM medium for 22–24 h followed by in vitro fertilization and culturing. The effect was evaluated by checking the cleavage rate at day-4 and the blastocyst development rate at day-8 post fertilization. As seen in Figure 1A, melatonin significantly enhanced the total cleavage rate that reached 79.75 ± 1.65 and 80.0 ± 3.48% when applied at concentrations 10^−9^ and 10^−8^ M respectively compared to the untreated control that showed 72.75 ± 1.54% cleavage rate (*p* ˂ 0.05). Although a slight decrease in the cleavage rate was observed in the group treated with 10^−7^ M melatonin (71.5 ± 1.32%), this effect was not statistically significant (*p* ˃ 0.05). Similarly, checking the day-8 blastocyst revealed significant improvement in melatonin-treated groups that gave 34.0 ± 2.27 and 34.5 ± 2.78% blastocyst rates for both 10^−9^ and 10^−8^ M groups compared to the control group that gave 26.7 ± 2.13% (Figure 1A). The 10^−7^ M melatonin concentration showed 27.7 ± 2.13% blastocyst development rate nonetheless did not reach the statistical significance.

In similar experimental settings, bovine oocytes were subjected to 22–24 h incubation with the AKT inhibitor SH6 during IVM step. Three concentrations were used including 25, 50 and 75 µM in addition to the control group that was left untreated. Microscopic investigation at day-4 revealed a strong inhibitory effect of SH6 on the total cleavage rate in a dose dependent manner (Figure 1B). The cleavage rates showed statistical significance when SH6 was administered at 50 and 75 µM compared to the control group (28.5 ± 3.59, 42.5 ± 2.5 and 63.5 ± 4.29 for 75, 50 and 25 µM, respectively). Although the lowest concentration of SH6, 25 µM, showed a reduction in the cleavage rate at day 4, this effect was statistical non-significant (*p* ˃ 0.05). Counting the total number of blastocysts developed at day-8 post fertilization showed dramatic drop upon addition of SH6 (Figure 1B). The highest concentration of SH6, 75 µM, resulted in 3.25 ± 0.629% blastocyst development rate compared to the 50 and 25 µM that gave 9.0 ± 1.68% and 16.5 ± 1.32, respectively.

### 2.2. Melatonin Addition During IVM Antagonizes the Anti-Developmental Effect of SH6

**Figure 2:**
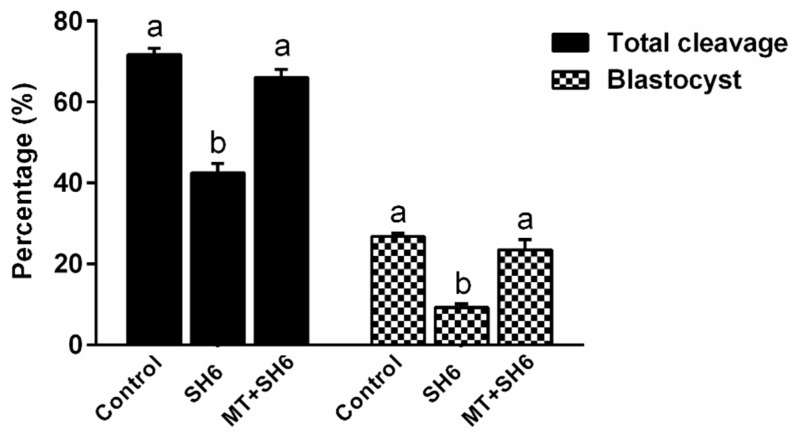
Effect of melatonin treatment during IVM on the development of SH6-treated oocytes. Oocytes were in vitro matured during exposure to 50 µM of SH6 in the presence or absence of melatonin (10^−8^ M) for 22–24 h. Data are expressed as mean ±SEM. Values with different superscripts indicate statistical significance (*p* < 0.05).

Since significant effects on embryo development were observed upon use of melatonin at concentrations 10^−8^ and 10^−9^ M, we first tested these two concentrations in combination with 50 µM of SH6 as it achieved nearly 50% reduction in the overall embryonic development process (half maximal effective concentration; EC_50_). From the initial microscopic examination, a significant positive effect of melatonin was achieved in the combination group only when administered at 10^−8^ M (data not shown). Therefore, all the following experiments were performed using the two fixed concentrations 10^−8^ M and 50 µM corresponding to melatonin and SH6, respectively. As seen in Figure 2, addition of SH6 during IVM step gave 42.5 ± 2.32% cleavage compared to the 71.75 ± 1.54% cleavage rate of the control. Interestingly, co-incubation with melatonin significantly improved the cleavage that reached 66 ± 2.04%. The lowest percentage of embryos developed to day-8 blastocyst was recorded in the SH6-treated group (9.25 ± 0.94%) which was significantly increased to 23.5 ± 2.53% upon addition of melatonin (Figure 2).

### 2.3. SH6 Treatment Adversely Affects, While Melatonin Rescues, the Nuclear Maturation and Cumulus Cell Expansion

**Figure 3:**
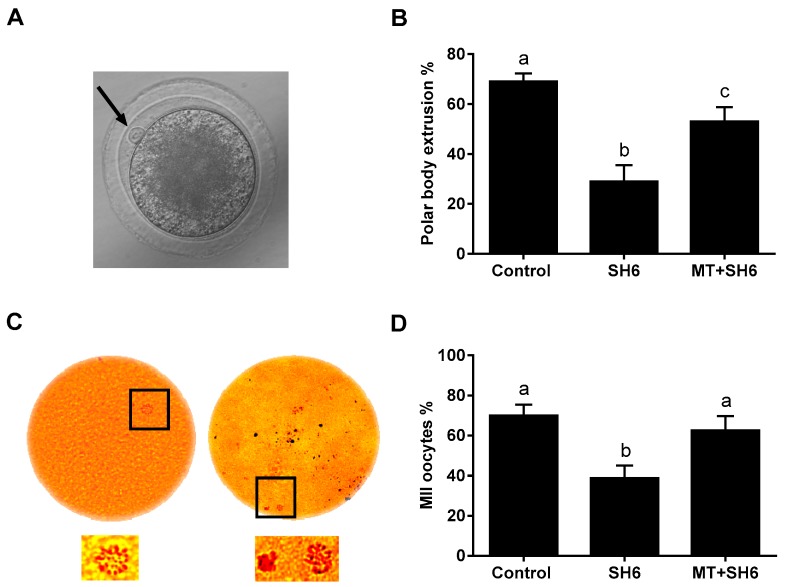
Microscopic determination of oocyte maturation using direct counting of polar body under light microscope or after aceto-orcein staining. (**A**) Appearance of first polar body (Black arrow) in in vitro matured oocyte under stereomicroscope; (**B**) Percentage of oocytes with obvious polar body. For confirmation, oocytes stained with aceto-orcein before examination under epifluorescence microscope; (**C**) The appearance of MI (to the left) and MII (to the right) stages oocytes. The lower parts are zooming for the area labeled with the black squares; (**D**) Percentage of MII-stage oocytes. Original magnification 200×.

As an indicator for oocyte maturation, MII-stage oocytes, the first polar body extrusion and the sequential expansion of cumulus cells were investigated 22–24 h after the onset of maturation of immature oocytes. Counting oocytes with visible polar body revealed a significant decline in SH6-treated group (29 ± 2.91%) compared to the SH6-melatonin co-treatment and control (53 ± 2.54% and 69 ± 3.31% respectively; Figure 3A,B). Aceto-orcein staining was applied for further confirmation of the MII-oocytes. As seen in Figure 3C,D, the percentage of MII-oocytes was significantly higher in melatonin-SH6 and control compared to the SH6-treated oocytes (62.5 ± 7.21%, 70 ± 5.4% and 38.75 ± 6.25% respectively). On the other hand, no statistically significant difference was observed between the melatonin-SH6 dual treatment and the control group (*p* ˃ 0.05).

**Figure 4:**
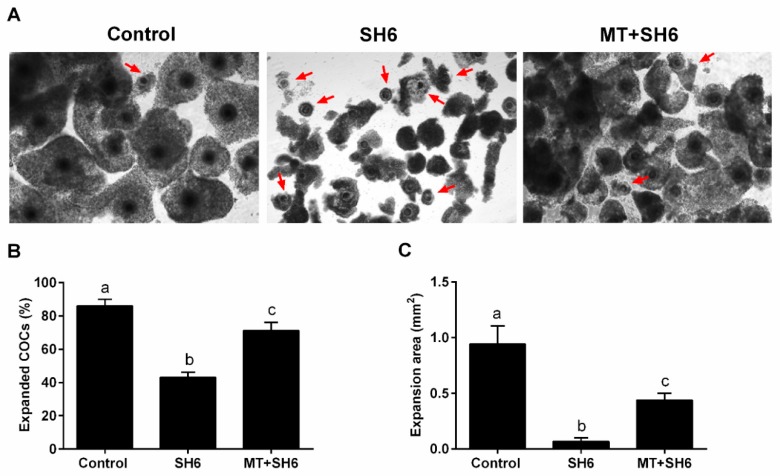
Morphological determination of cumulus cells expansion during in vitro maturation. (**A**) Light microscopy images of day 1 cumulus oocyte complexes (COCs) matured in vitro. Red arrows indicate non-expanded COCs; (**B**) Quantification of expanded COCs; (**C**) The increase in the expansion area of COCs. Different superscript letters indicate statistically significant difference (*p* < 0.05). Original magnification 40×.

At the end of maturation, the number of expanded COCs and the degree of expansion of individual COCs were recorded. As seen in Figure 4A, a clear morphological difference was observed regarding cumulus cell expansion that showed lowest level in the SH6-treated group. The percentage of expanded COCs was lower in the SH6-treated than the co-treated and the control groups (43.05 ± 3.04%, 71.12 ± 5.01% and 85.95 ± 2.06% respectively; Figure 4B). In parallel, measuring the degree of expansion showed higher values in both melatonin-SH6 co-treated and control COCs compared to SH6 ones (Figure 4C).

**Figure 5:**
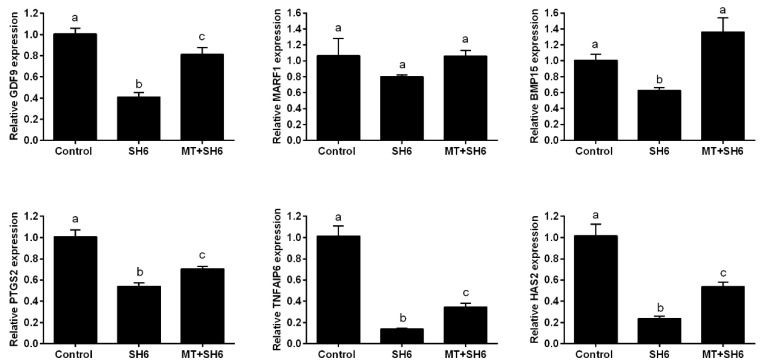
Relative expression of the mRNA of genes involved in oocyte maturation and cumulus cells expansion in COCs. Superscripts with different letters indicate statistical significance (*p* < 0.05).

For molecular characterization of oocyte maturation process, the mRNA levels of the maturation-related genes GDF-9, MARF1, and BMP-15 were quantified by RT-qPCR. High expression levels were observed in both melatonin-SH6 dual treatment and the control groups compared to SH6 (Figure 5). Similarly, RT-qPCR analysis of cumulus expansion-related genes including PTGS2, TNFAIP6 and HAS2 showed down-regulation of the mRNA levels in SH6 treated group that were partially restored upon addition of melatonin (Figure 5).

### 2.4. Melatonin Reduces the Oxidative Stress and Apoptosis in SH6-Treated Oocytes

**Figure 6:**
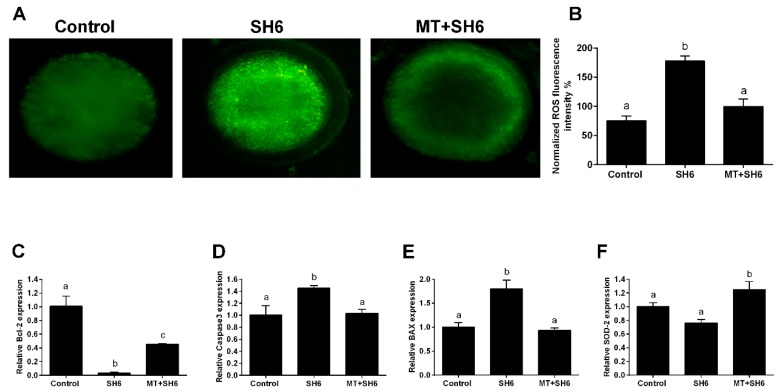
Determination of oxidative stress and apoptosis using ROS assay and RT-qPCR. (**A**) Microscopic determination of matured live oocytes stained with H_2_DCFDA as an indicator for intracellular ROS concentration in control and treated groups; (**B**) Fluorescent intensity analyzed by ImageJ software; (**C**–**F**) Relative expression of the mRNA of oxidative stress and apoptosis-related genes. Superscripts with different letters indicate statistical significance (*p* < 0.05). Original magnification 200×.

**Figure 7:**
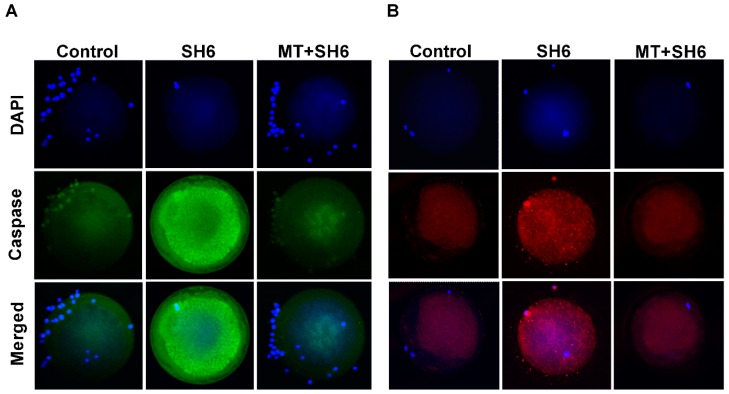

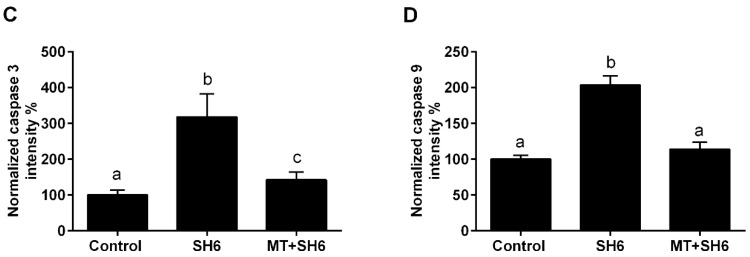
Immunofluorescence on matured oocytes showing caspase 3 (**A**) and caspase 9 (**B**) expression; (**C**,**D**) Mean values of the integrated optimal densities of caspase 3 and caspase 9, respectively. Superscripts with different letters indicate statistical significance (*p* < 0.05). Original magnification 100×.

To inspect the oxidative stress in oocytes, intracellular ROS levels were estimated using 2,7-dichlorodihydrofluorescein diacetate (H_2_DCFDA) staining. The highest fluorescence intensity, an indicator for the ROS level, was observed in SH6-treated group compared the control and melatonin-SH6 co-treated groups (Figure 6A,B). Furthermore, SH6 treatment resulted in a significant down-regulation of the mRNA level of the anti-apoptotic gene Bcl-2 and a slight down-regulation of the antioxidant gene SOD-2 whereas the apoptosis-related genes BAX and caspase 3 were significantly up-regulated (Figure 6C–F). In parallel, addition of melatonin during SH6 exposure up-regulated the mRNA level of SOD-2 and Bcl-2 while down-regulated the BAX and caspase 3 (Figure 6C–F). Immunofluorescence using antibodies specific to caspase 3 and caspase 9 showed high expression levels of the two proteins in SH6-treated group compared to combination and control groups (Figure 7).

### 2.5. Melatonin Normalizes DNA Stability, Mitochondrial Activity, and Cell Cycle Following SH6 Exposure

**Figure 8:**
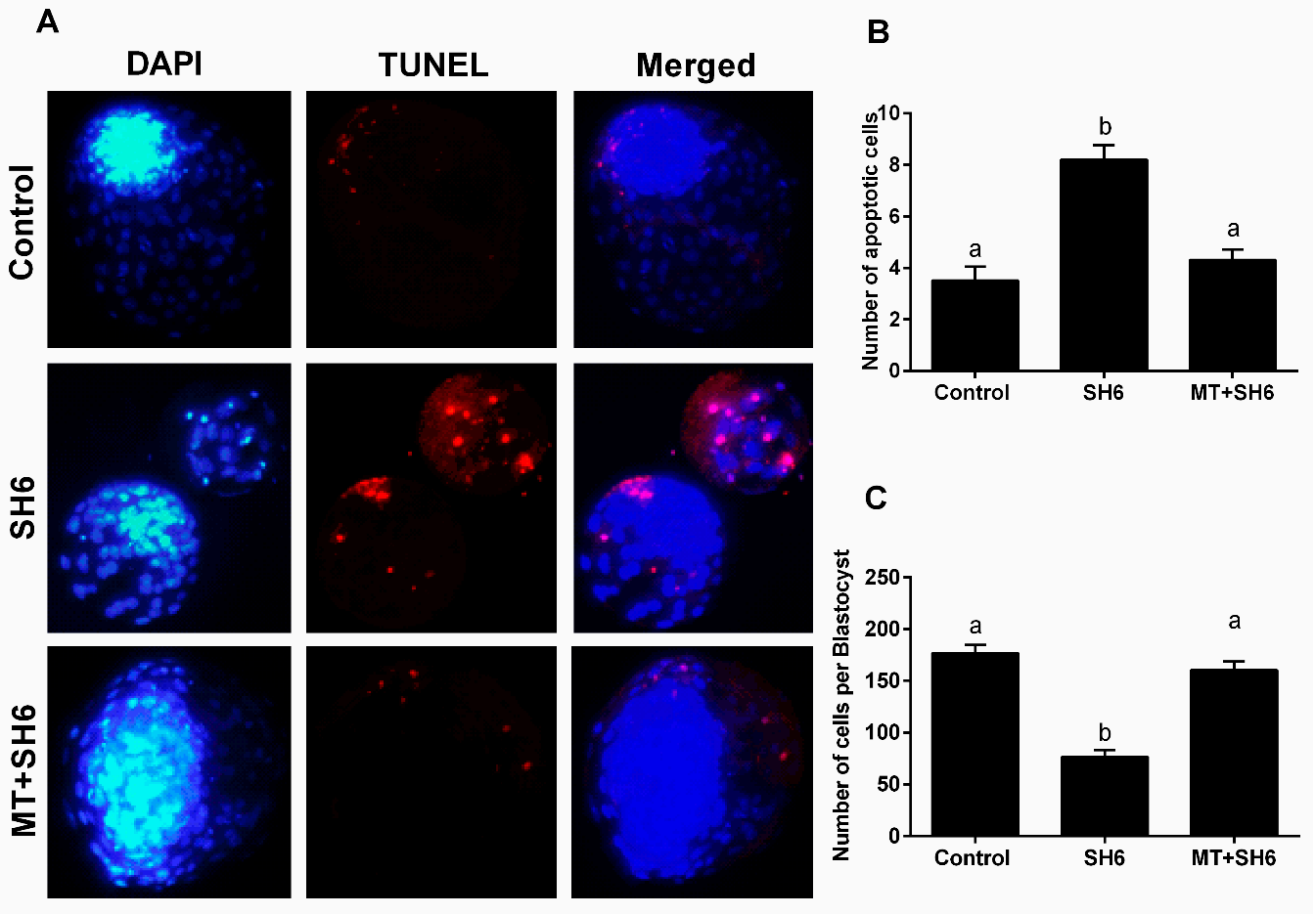
Effect of melatonin supplementation during SH6 exposure on the quality of embryos. (**A**) Nuclei were stained using DAPI while fragmented DNA was probed by TUNEL (**B**) Number of apoptotic cells per blastocyst; (**C**) Total number of cells per blastocyst. Values with different superscripts indicate significant differences (*p* < 0.05). Original magnification 200×.

To assess the possible DNA fragmentation inside embryos, Transferase dUTP Nick End Labeling (TUNEL) assay was applied. Lower numbers of apoptotic cells were recorded in both control and melatonin-SH6 co-treated groups (3.45 ± 0.49 and 4.3 ± 0.42) compared to the high incidence of apoptosis observed upon SH6 sole treatment (8.2 ± 0.55; Figure 8A,B). The total number of cells per blastocyst counted using 4′,6-diamidino-2-phenylindole (DAPI) staining was significantly lower in SH6 group than melatonin-SH6 and control groups (76.14 ± 6.84 vs 160 ± 8.22 and 176.25 ± 8.26, respectively; Figure 8C) (*p* < 0.05).

**Figure 9:**
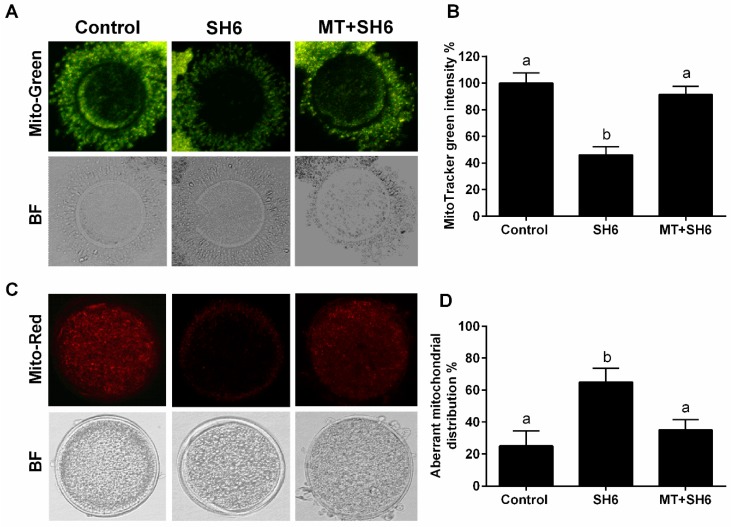
Effect of melatonin supplementation on mitochondrial membrane potential and mitochondrial distribution pattern in matured oocytes. (**A**) MitoTracker green fluorescence staining; (**B**) Integrated optical density; (**C**) Representative images of MitoTracker Red staining in matured oocytes; (**D**) Proportion of oocytes with aberrant mitochondrial distribution in each treatment group. BF: Bright Field. Values with different superscripts indicate significant differences (*p* < 0.05). Original magnification 200×.

The impact of SH6 and melatonin treatment on mitochondrial membrane potential (MMP) and mitochondrial distribution pattern in the in vitro matured oocytes was investigated using MitoTracker Green and MitoTracker Deep Red, respectively. As seen in Figure 9A,B, a significant reduction in the integrated mitochondrial fluorescence intensity was observed in SH6-treated group compared to melatonin-exposed and control groups. Regarding mitochondrial distribution, higher incidence of aberrant pattern was observed in SH6-treated oocytes than the other two groups (Figure 9C,D).

**Figure 10:**
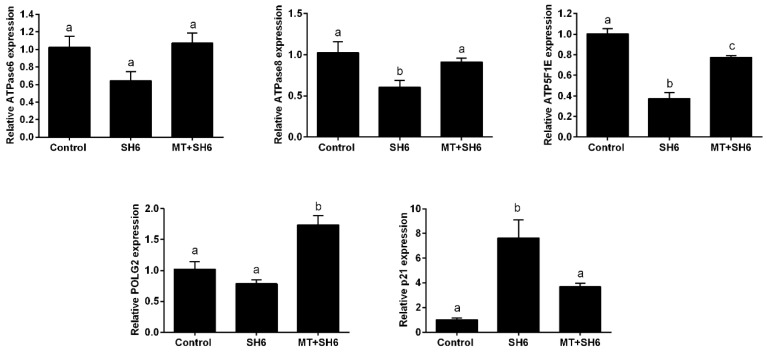
Relative expression of the mRNA of different mitochondrial and cell cycle-related genes. Superscripts with different letters indicate statistically significance (*p* < 0.05).

Moreover, the RT-qPCR of the mitochondrial-related genes ATPase6, ATPase8, ATP5F1E, and POLG2 showed down-regulation in the SH6-treated oocytes while the opposite profile was observed in melatonin-treated group (Figure 10). In addition, testing the mRNA level of p21 involved in cell cycle arrest showed an increase following SH6 treatment but decreased upon addition of melatonin (Figure 10).

### 2.6. Potential Interplay between Melatonin and AKT Signaling

**Figure 11:**
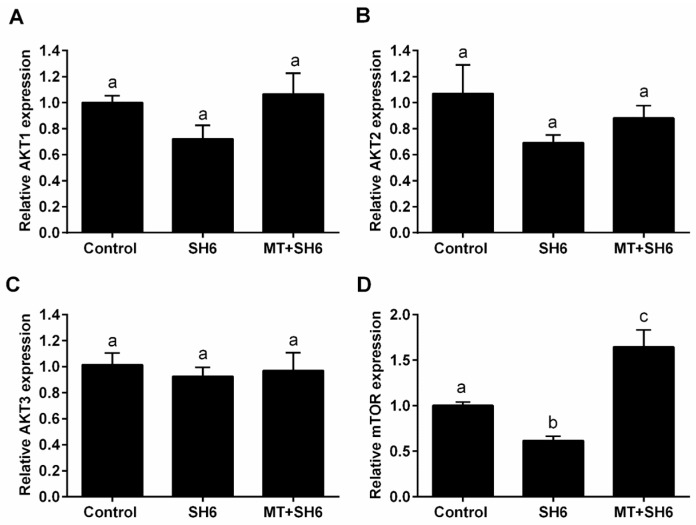
Relative expression of the mRNA of AKT signaling-related genes in oocytes. The mRNA from melatonin-SH6, SH6, and control groups was used for relative quantification of AKT1 (**A**), AKT2 (**B**), AKT3 (**C**), and mTOR (**D**). Superscripts with different letters indicate statistical significance (*p* < 0.05).

To elucidate the possible mechanisms underlying the protective role of melatonin against the AKT inhibitor SH6, the mRNA levels of AKT1, AKT2, AKT3, and mTOR, and the protein levels of total AKT and phosphorylated AKT (tAKT and pAKT) were investigated in in vitro matured oocytes following exposure to SH6 in presence and absence of melatonin by RT-qPCR and immunofluorescence respectively. A slight down-regulation of the mRNA of AKT1, AKT2 and AKT3 was observed following SH6 treatment compared to the control and combination groups but this did not reach the statistical significance (Figure 11A–C). The mTOR was down-regulated in SH6-treated oocytes while up-regulated following melatonin administration (Figure 11D).

**Figure 12:**
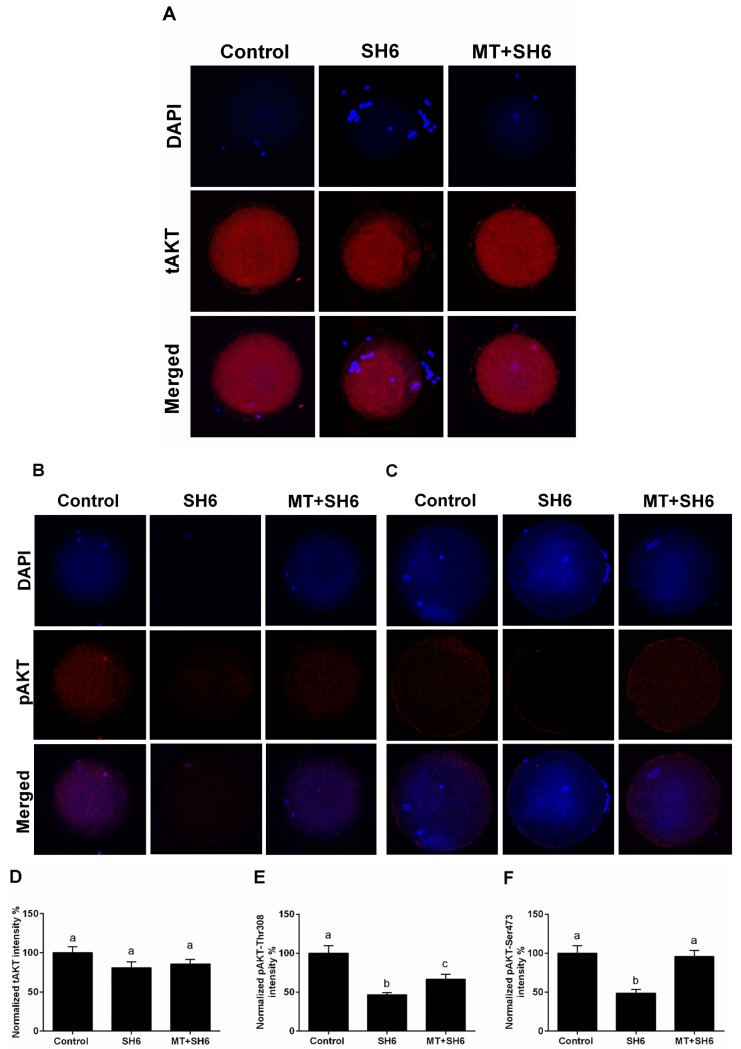
Immunofluorescence of total and phosphorylated AKT in oocytes. (**A**) Total AKT; (**B**,**C**) Phosphorylated AKT-Thr308 and AKT-Ser473 respectively; (**D**–**F**) Mean values of the integrated optimal densities of total and phosphorylated AKT. tAKT: total AKT; pAKT: phosphorylated AKT. Superscripts with different letters indicate statistical significance (*p* < 0.05). Original magnification 100×.

In addition, immunofluorescence using antibodies raised against total (tAKT) and phosphorylated AKT (pAKT-Thr308 and pAKT-Ser473) showed low levels of pAKT in SH6-treated oocytes compared to melatonin co-treated and control groups while comparable levels of the total AKT were observed across all groups (Figure 12).

## 3. Discussion

The relatively high levels of melatonin in the follicular fluid and the presence of melatonin receptors on the surface of oocytes and cumulus cells [43] in addition to the ability of oocyte itself to synthesize this multitasking molecule highlight its potential role in oocyte life span and the developmental competence of embryos [44]. It has been shown that melatonin protects oocytes and the developing embryos from the oxidative stress and nuclear fragmentation [45,46,47]. Melatonin also alleviated the apoptotic effect of mycotoxins (β-zearalenol and HT-2) in bovine ovarian granulosa cells [37]. Moreover, it protected oocytes from the harmful effect of paraquat (a widely used herbicide), aflatoxin B1 and Bisphenol A [34,35,36]. However, the actual mechanisms underlying this function are not fully clarified. One possible mechanism is related to the direct function of melatonin as a potent antioxidant via scavenging the ROS and hence mitigating the pressure of oxidative stress in oocytes and developing embryos. Another possibility is the potential involvement of melatonin in regulation of certain cellular signaling pathways such as PI3K/AKT/mTOR that could positively affect the maturation of oocytes and the subsequent embryonic progress.

On the other hand, high concentration of active AKT, mainly the pAKT-Ser 473, in more than 90% of in vivo and in vitro matured oocytes has been reported, whereas studies have clarified the functional role of AKT during oocyte maturation and subsequent embryo development [13,17,38,48]. Although the interaction between melatonin and AKT signaling was previously recorded in some cells such as brain astrocytes [41], until now there is no data on a possible linkage in mammalian oocytes. Therefore, we started deciphering the possible interaction between melatonin and AKT signaling in bovine oocytes through studying the protective role of melatonin against the AKT inhibitor (SH6)-induced apoptosis during oocyte in vitro maturation. The phosphatidylinositol analogue SH6 is a cell permeable substrate that inhibits the AKT phosphorylation without affecting total AKT abundance [17]. Our initial data revealed that 10^−9^ and 10^−8^ M of melatonin significantly improved the cleavage and blastocyst rates confirming the previous reports [34,49]. Contrarily, SH6 clearly abrogated the cleavage and the percentage of developed blastocysts in a dose dependent manner whereas 50 μM of SH6 achieved approximately 50% inhibition. These results are in line with previous studies that used LY294002 and SH6 as inhibitors for PI3K/AKT signaling and showed a significant decrease in the total cleavage and blastocyst development [17,50].

Surprisingly, the addition of melatonin during SH6 treatment dramatically barricaded the anti-developmental effect of AKT inhibition during oocyte maturation and eventually improved the nuclear maturation, cumulus cells expansion, early cleavage, and more importantly the day-8 blastocyst development rate. We went further to test the quality of oocytes and embryos following melatonin co-administration during SH6 exposure. The total cell number per each blastocyst was significantly boosted upon addition of melatonin to the SH6-treated group that reached a level comparable to control. Testing the DNA fragmentation inside embryos using TUNEL assay showed lower number apoptotic cells in both control and melatonin-SH6 treated blastocysts compared to the more apoptotic cells observed in SH6-treated ones. During in vitro maturation, the degree of expansion of cumulus cells normally reflects the quality of oocyte maturation process and could predict the competency of future embryos [49]. In the present study, morphological examination of COCs showed higher degree of expansion in melatonin-SH6 co-treated group compared to the diminished expansion observed in SH6-solely treated COCs. Testing the expression levels of different cumulus expansion-related genes showed down-regulation of HAS2, TNFAIP6, and PTGS2 after SH6 treatment while the levels were partially normalized by melatonin. This was comparable to the morphological profiles and also with previous reports showing the positive effect of melatonin on oocyte maturation through promoting cumulus expansion [49,51].

In line with cumulus results, the maturation-related genes including GDF-9, BMP-15 and MARF1 exhibited lower expression levels in SH6-treated oocytes while melatonin administration rescued the normal levels in case of GDF-9 and BMP-15. This highlights the beneficial role of melatonin, especially under the stress of AKT inhibitors and anti-developmental compounds, for oocyte maturation since previous studies showed the essential role of GDF-9, MARF1 and BMP-15 for the developmental competence of oocyte [47,52,53]. This also supports our data on the polar body visualization, used as an indicator for successful maturation, that showed higher MII-stage percentage in melatonin-SH6 co-treated oocytes compared to SH6. Moreover, these observations were significantly supported by the results of TUNEL and the total number of cells per blastocyst reflecting the positive effect of melatonin on improving the quality of developing embryos especially under the pressure of cytotoxic compounds [49,51].

Due to the crucial role of mitochondrial activity in oocyte maturation and the competency of developing embryos, we went further for testing the expression levels of different mitochondrial-related genes including ATPase6, ATPase8, ATP5F1E, and POLG2. Data showed down-regulation of these genes in SH6-treated oocytes only while up-regulated upon addition of melatonin similar to previous melatonin studies [32,54,55,56]. Altogether, this provides concrete evidence for the direct involvement of melatonin in maturation and meiosis regulation in bovine oocytes.

One of the most challenges facing oocyte maturation process is the ROS excessive production associated with oxidative stress which consequently diminishes the proper oocyte maturation and embryo development [57]. Using ROS measurement assay, we found that melatonin successfully reduced the ROS levels under the pressure of AKT inhibition. This reflects the role of melatonin in reducing the oxidative stress in bovine oocytes. For more confirmation, the intensity of MMP was measured. The MMP is an essential component in the process of energy storage during oxidative phosphorylation and it reflects the normal oocyte physiological activity. Since the decrease in MMP could be an obvious indicator for mitochondrial damage and the early phase of apoptosis [58], here we found that AKT inhibition dramatically reduced the MMP in oocytes, an effect that was subsequently neutralized upon co-administration of melatonin. Additionally, we noticed a clear reduction in the normal mitochondrial distribution pattern upon SH6 treatment, while melatonin efficiently displayed the normal distribution. This confirms the previous studies which reported the importance of melatonin for maintaining the optimal mitochondrial function by reducing the oxidative stress [59,60,61]. It also corroborates our above-mentioned records on oocyte maturation and blastocyst development where a significant decreased level of apoptosis in melatonin-SH6 co-treated group was observed compared to the high apoptosis under SH6 stress. This was further clarified at molecular level using RT-qPCR that showed a slight down-regulation in SOD-2 in SH6-treated oocytes while a significant up-regulation was observed after melatonin administration.

Detection of AKT at genetic and protein levels in SH6-treated oocytes showed slight decrease upon addition of SH6 whereas a significant effect was observed in the case of phosphorylated AKT. Interestingly, the addition of melatonin induced activation of AKT as seen in the higher levels of pAKT-Ser473 and pAKT-Thr308 observed in melatonin-SH6 co-treated oocytes reflecting the potential interplay between melatonin and AKT signaling pathway in the favor of oocyte maturation and embryo development. These results are in line with the previous relationship between melatonin and AKT signaling reported in astrocytes and theca cells [41,42]. It was shown that AKT has a vital role in stimulating the transition of cell cycle to higher proliferation through regulation of other target proteins involved in cell cycle control such as p21 and p27 [7,62,63]. Since the reduction of AKT phosphorylation previously delayed cell cycle progression [64], we checked the expression level of p21 as a cell cycle arrest indicator. Our results showed that the expression level of p21 was dramatically increased upon SH6 treatment which reflects the harmful effect of AKT silencing on oocyte cell cycle. Obviously, melatonin supplementation during the pressure of SH6 decreased the expression level of p21. This is in line with Spencer et al. who reported the failure of cell proliferation under high p21 level and affirms the cytoprotective function of melatonin [65].

Through inhibition of tuberous sclerosis protein 2 (TSC2), the AKT also activates mTOR which functions as serine/threonine protein kinase regulating cell growth, survival, protein synthesis and autophagy [66]. The mTOR is also expressed in all stages oocytes showing a role in meiosis completion and embryonic development [67]. Our gene expression analysis showed up-regulation of the mTOR level upon melatonin co-treatment while down-regulation was observed following AKT inhibition. This might corroborate the activity of melatonin and AKT in oocyte for induction of competent maturation. Moreover, one of the common cell survival protective mechanisms mediated by AKT is through suppressing the level of different pro-apoptotic mediators including Bcl-2 associated death promotor (BAD) and caspase 9 [18,19,20]. Our data showed higher mRNA and protein levels of the pro-apoptotic markers BAX, caspase 3 and caspase 9 in SH6 treated oocytes while the Bcl-2 decreased but the opposite profile was recorded in case of melatonin supplementation. Matching with our finding, the BAX/Bcl-2 ratio, used as an indicator for apoptosis, decreased upon treatment with melatonin, the effect that was counteracted by addition of selective melatonin receptor antagonists [51].

**Figure 13:**
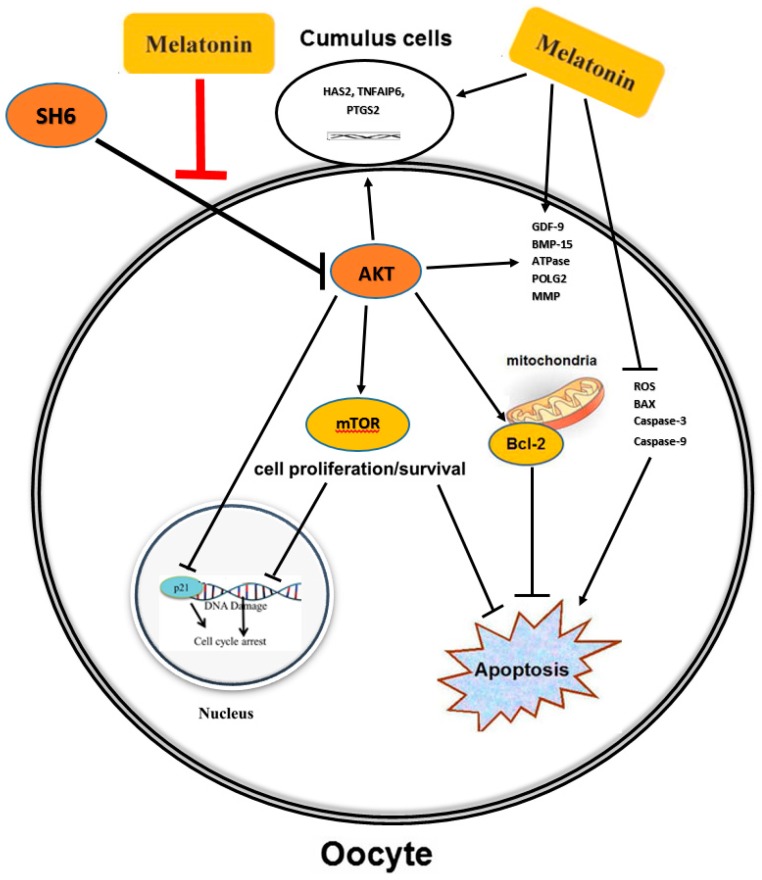
Proposed mechanism of action of melatonin in bovine oocyte under SH6 pressure. Melatonin protects oocyte and efficiently improves the developmental competence of embryos through different pathways. The first is based on the direct antioxidant and anti-apoptotic function of melatonin under SH6 exposure. The other is the potential interaction with the AKT activation pathway that positively increases the maturation rate of oocytes and subsequent developmental processes.

In conclusion, we report that the antioxidant activity of melatonin improves the embryo development through enhancement of oocyte maturation, cumulus cells expansion, and protection from DNA fragmentation under the stress of AKT inhibition. Furthermore, the activity of melatonin makes it a potentially useful tool in assisted reproductive technology especially in the case of inferior low-quality oocytes. The protection against the consequences of AKT inhibition observed upon administration of melatonin suggests a possible linkage between this pineal hormone and the AKT signaling in bovine oocyte (Figure 13). This paves the way for more in-depth studies on melatonin-AKT interplay throughout the entire pre-implantation embryo development processes.

## 4. Materials and Methods

### 4.1. Ethics Statement, Reagents, and Experimental Design

Study experiments were performed according to the Guidelines of Gyeongsang National University (Approval ID: GAR-110502-X0017; date 02-05-2011) under the regulation of the Institutional Animal Care and Use Committee. Reagents used were purchased from Sigma-Aldrich (St. Louis, MO, USA) unless otherwise stated. The experimental work of the current study underwent through three major steps. We first tested the effect of melatonin on bovine oocyte maturation and embryo development when added to IVM medium. Three different concentrations of melatonin (10^−9^, 10^−8^ and 10^−7^ M) were used in addition to an untreated control group while the day-4 cleavage and day-8 blastocyst development rates were recorded. Step 2 was carried out to study the impact of the AKT inhibitor SH6 on developmental process of bovine embryos when used at concentrations 25, 50 and 75 μM. The third step included SH6-treated group (50 μM), melatonin-SH6 combination treatment group (50 μM and 10^−8^ M for SH6 and melatonin respectively) and a control group that was left untreated. In all experiments, oocytes were plated at density around 50 oocytes per well in 4 replicates.

### 4.2. Oocyte Collection and IVM

Bovine ovaries were collected from a local abattoir in South Korea, transported to laboratory within 2 h after slaughter and washed in fresh pre-warmed Dulbecco’s phosphate-buffered saline (DPBS). The COCs were aspirated from 2–8 mm diameter follicles in TL-HEPES medium (114 mM sodium chloride, 2 mM sodium bicarbonate, 0.34 mM sodium biphosphate, 10 mM sodium lactate, 3.2 mM potassium chloride, 0.5 mM magnesium chloride, 2.0 mM calcium chloride, 10 mM HEPES, 1 μL/mL phenol red, 0.1 mg/mL streptomycin and 100 IU/mL penicillin) using 18-gauge needle attached to a vacuum pump. Oocytes with at least three layers of compact cumulus cells and homogenous cytoplasm were picked up under stereomicroscope (Olympus SZ51, Tokyo, Japan). The collected oocytes were washed thrice in IVM medium (TCM-199 supplemented with 1 μg/mL estradiol-17β, 10 μg/mL follicle-stimulating hormone (FSH), 10 ng/mL epidermal growth factor (EGF), 0.6 mM cysteine and 0.2 mM sodium pyruvate and 5% fetal bovine serum (FBS; Gibco BRL, Life Technologies, Grand Island, NY, USA)). Oocytes were cultured in 700 μL of IVM medium in four-well plates (Thermo Fisher Scientific, Waltham, MA, USA) in presence or absence of SH6 and melatonin then incubated at 38.5 °C and 5% CO_2_ for 22–24 h.

### 4.3. In Vitro Fertilization (IVF) and Embryo Development

Following in vitro maturation, oocytes were fertilized with cryopreserved bovine sperm as previously mentioned [30]. Briefly, frozen semen was immediately thawed at 38 °C for 1 min and diluted in warm DPBS followed by centrifugation at 750× *g* for 5 min at room temperature. The supernatant was aspirated and the sperm-containing pellet was re-suspended in 500 μL heparin (20 μg/mL) prepared in IVF medium (Tyrode’s lactate solution, 6 mg/mL bovine serum albumin (BSA), 22 mg/mL sodium pyruvate, 0.1 mg/mL streptomycin and 100 IU/mL penicillin) and incubated at 38.5 °C for 15 min. Concentrated sperm were diluted with IVF medium to a final density of 1 × 10^6^ sperm/mL, yet 700 μL was added to each oocytes group. Oocytes-sperm mixtures were incubated for 18–20 h at 38.5 °C and 5% CO_2_ then the cumulus cells were removed by successive pipetting. After washing with SOF-BE1 medium supplemented with 4 mg/mL BSA, 5 μg/mL insulin, 5 μg/mL transferrin and 5 ng/mL sodium selenite (SOF+BSA+ITS), presumptive zygotes were transferred to 4-well plates containing 700 μL SOF+BSA+ITS medium and kept at 38.5 °C under 5% CO_2_. Three days later, the total cleavage was recorded then the 8-cell stage embryos were further incubated in fresh medium for another 4 days. Day-8 blastocysts (day 0= day of IVF) were washed three times in TL-HEPES and either transferred to 4% paraformaldehyde prepared in phosphate-buffered saline (PBS) and stored at 4 °C or snap frozen in liquid nitrogen and kept at −80 °C until use.

### 4.4. Cumulus Expansion Assessment

To evaluate the in vitro maturation of oocytes, the percentage of morphologically expanded COCs at the end of maturation (triplicates; n = 50) was estimated using stereomicroscope. In addition, individual COCs (triplicate; n = 15–20) were examined under epifluorescence microscope (Olympus, IX71) before and after maturation. The area of expansion (mm^2^) was calculated using ImageJ software (National Institutes of Health, USA; https://imagej.nih.gov/ij/).

### 4.5. Assessment of Oocyte Maturation by Aceto-Orcein Staining and First Polar Body Extrusion

The percentages of MII-stage oocytes were determined by direct microscopic examination of the polar body and by aceto-orcein staining. Briefly, the in vitro matured COCs were denuded of the surrounding cumulus cells by gentle vortex in 0.1% (w/v) hyaluronidase for 2–3 min. As an indicator for nuclear maturation, denuded oocytes (5 replicates; n = 20) were examined under stereomicroscope for the presence of the first polar body. For aceto-orcein staining, denuded oocytes were fixed in methanol and acetic acid mixture (3:1) for at least 24 h then stained with 1% aceto-orcein (prepared in 45% acetic acid) and examined by epifluorescence microscope. The stage of the nuclear maturation was determined according to the morphology of the nuclear material. Oocytes were classified as: metaphase I stage (MI, immature oocytes) or metaphase II (MII, mature oocytes). Data analysis was performed after 3 times replicate (n = 25–30 oocytes per replicate).

### 4.6. Measurement of Intracellular ROS Level

The 2,7-dichlorodihydrofluorescein diacetate (H_2_DCFDA) was used to detect intracellular ROS level. In brief, in vitro matured oocytes (triplicates; n = 15–20) were treated with H2DCFDA (10 μM in PBS-PVA) and incubated for 20 min at 38.5 °C and 5% CO_2_. After washing three times with PBS-PVA, oocytes were spotted on glass slide and examined using epifluorescence microscope under 490 nm excitation and 525 nm emission wavelengths. For image analysis, fluorescence intensity was analyzed using ImageJ software.

### 4.7. MMP and Mitochondrial Distribution Pattern

To investigate the mitochondrial membrane potential, in vitro matured oocytes (triplicates; n = 10–15) were subjected to Mito Tracker Green assay according to the manufacturer’s instructions (Invitrogen, Carlsbad, CA, USA). Briefly, partially denuded oocytes were stained with 125 nM MitoTracker Green FM for 30 min followed by washing with PBS-PVA. Oocytes mounted on glass slides were examined immediately using inverted epifluorescence microscope and the fluorescence intensity were analyzed using ImageJ software. MitoTracker deep Red FM (Molecular Probes, Eugene, OR, USA) was used to determine mitochondrial distribution pattern in in vitro matured oocytes [45]. Briefly, oocytes were incubated with 100 nM MitoTracker for 40 min at 38.5 °C then fixed in 4% paraformaldehyde for 15 min at 38.5 °C after washing in PBS-PVA. Oocytes were spotted on glass slides under cover slips and examined using inverted epifluorescence microscope. The mitochondrial distribution patterns were classified either as homogeneous where the mitochondria are dispersed throughout the cytoplasm or as aberrant pattern which shows mitochondrial distribution in cytoplasm either peripheral or semi peripheral pattern located beneath the plasma membrane.

### 4.8. Terminal Deoxynucleotidyl TUNEL Assay

For assessment of apoptotic level and embryotic quality, TUNEL assay was performed using In Situ Cell Death Detection kit (Roche Diagnostics, Indianapolis, USA) according to the manufacturer’s guidelines. Fixed blastocysts (triplicates; n = 15) were washed twice with 0.3% [*w*/*v*] polyvinylpyrrolidone (PVP) prepared in PBS and then permeabilized using 0.5% Triton X-100 and 0.1% sodium citrate for 30 min at room temperature. Blastocysts were then washed twice with PBS and incubated with fluorescent-conjugated terminal deoxynucleotide transferase dUTP for 1 h at 38.5 °C followed by washing with PBS-PVP. Embryos were incubated for 10 min with 1 µg/mL 4′,6-diamidino-2-phenylindole (DAPI) then mounted on glass slide. The nuclear configuration was analyzed using confocal laser-scanning Olympus Fluoview FV1000 microscope where the TUNEL-positive cells, specifying the level of apoptosis, appeared as bright red.

### 4.9. Total Ribonucleic Acid (RNA) Extraction and cDNA Synthesis

Total RNA was extracted from COCs (nearly 50 per group) using Arcturus PicoPure RNA Isolation Kit according to the manufacturer’s instructions (Arcturus, Foster, CA, USA). After incubation of columns with DNase for 15 min, the total RNA was eluted in 15 μL elution buffer. The RNA was immediately subjected to cDNA synthesis using iScript cDNA synthesis kit according to manufacturer’s instructions (Bio-Rad Laboratories, Hercules, USA). Briefly, 4 μL of 5X iScript reaction mixture and 1 μL of iScript reverse transcriptase were added to the 15 μL RNA then the cDNA was synthesized under the following reaction conditions: incubation at 25 °C for 5 min, 42 °C for 30 min and enzyme inactivation at 85 °C for 5 min. The cDNA concentration was determined using NanoDrop 2000c spectrophotometer (Thermo Fisher Scientific) and stored at −20 °C until use.

### 4.10. Quantitative Reverse Transcription Polymerase Chain Reaction (RT-qPCR)

The cDNA was subjected for RT-qPCR analysis using iQ-SYBR GREEN Supermix according to manufacturer instructions. Briefly, 3 μL of diluted cDNA were mixed with 2 μL of primer mixture and 5 μL iQ-SYBR GREEN Supermix. The primers used were either designed using primer3 online software (https://primer3plus.com/cgi-bin/dev/primer3plus.cgi) based on the mRNA sequences of certain bovine genes downloaded from the GenBank or from previous publications (Table 1). The qPCR reaction was performed using CFX98 instrument (Bio-Rad Laboratories) under the following conditions: initial denaturation at 95 °C for 3 min followed by 44 cycles of 95 °C for 15 s, 58 °C for 20 s and 72 °C for 30 s, and a final extension at 72 °C for 5 min. To improve the reproducibility of the results, each cDNA sample was run in duplicate for each PCR reaction. Melting curve analysis using progressive denaturation, during which the temperature increased from 65 °C to 95 °C at a rate of 0.2 °C per second was also applied. The full names of the genes used in the study are: cysteine-aspartic acid protease (Caspase 3), B-cell lymphoma 2 (Bcl-2), Bcl-2 associated X apoptosis regulator (BAX), superoxidase dismutase 2 (SOD-2), cyclin-dependent kinase inhibitor 1 (p21), protein kinase B (PKB or AKT), mammalian target of rapamycin (mTOR), growth differentiation factor-9 (GDF-9), bone morphogenetic protein 15 (BMP-15), meiosis regulator and mRNA stability factor 1 (MARF1), hyaluronan synthase 2 (HAS2), tumor necrosis factor alpha-induced protein 6 (TNFAIP6), prostaglandin-endoperoxide synthase 2 (PTGS2), adenylpyrophosphatase (ATPase6 and ATPase8), ATP synthase F1 subunit epsilon (ATP5F1E), mitochondrial-specific DNA polymerase gamma (POLG2), and glyceraldehyde-3-phosphate dehydrogenase (GAPDH). The abundance of each gene was relatively calculated using the ΔΔ*C*t method whereas the GAPDH was used as a housekeeping gene.

### 4.11. Immunofluorescence Analysis

Partially denuded COCs (20 oocytes per group) were fixed in 4% paraformaldehyde for 15 min then washed three times with PBS. Permeabilization was conducted by incubation with 0.5% Triton X-100 for 20 min followed by washing three times. Proteinase K was added, and plates were incubated for 5 min at room temperature before washing. Oocytes were incubated for 2 h at room temperature with blocking buffer (10% FBS and 3% BSA in PBS) before incubation overnight at 4 °C with the primary antibodies (diluted in 3% BSA and 0.1% Tween 20 in PBS). Antibodies targeting caspase 3 and caspase 9 (Santa Cruz Biotechnology, Santa Cruz, CA, USA), total AKT (Abcam), Phospho-AKT (Thr308 and Ser473; Cell Signaling Technology) have been used. After washing, fluorescein isothiocyanate (FITC)- and tetramethylrhodamine isothiocyanate (TRITC)-conjugated secondary antibodies (Santa Cruz Biotechnology; diluted 1:100 in PBS) were added and plates were incubated 90 min at room temperature. The nuclei were stained with DAPI for 10 min followed by washing three times. The stained oocytes were spotted on glass slides, visualized under a confocal laser-scanning Olympus Fluoview FV1000 microscope, yet the optical densities were determined using ImageJ.

### 4.12. Statistical Analysis

The data presented in this study were analyzed using GraphPad Prism software version 6. Differences in all embryonic development process and transcription levels of studied genes among the study groups were analyzed using one-way ANOVA followed by Duncan’s multiple comparison test. All images presented were analyzed using ImageJ software. All values were presented as the mean values ± standard error (SEM) and the *p*-values below 0.05 were considered statistically significant.

## Figures and Tables

**Table 1:** Genes names, accession numbers, the sequences of primers used in RT-qPCR, and sizes of PCR products.

Gene Name	Sequence	GenBank Accession Number	Product Size (bp)
Caspase 3	F: CCCAAGTGTGACCACTGAAC R: CCATTAGGCCACACTCACTG	NM_001077840	169
BAX	F: CACCAAGAAGCTGAGCGAGTGT R: TCGGAAAAAGACCTCTCGGGGA	NM_173894	118
Bcl-2	F: TGGATGACCGAGTACCTGAA R: CAGCCAGGAGAAATCAAACA	NM_001166486	120
SOD-2	F: GGGAGAATGTAACTGCACGA R: ACAACAGAGCAGCGTACTGG	NM_201527.2	133
GDF-9	F: GGACCCCTAAATCCAACAGA R: ACAGTAACACGATCCAGGTT	NM_174681.2	123
BMP-15	F: TCCAGAACCTTGTCAATGAG R: GGGCAATCATACCCTCATAC	NM_001031752.1	141
MARF1	F: GCAGAGCACCAGGACAATCA R: GAAATAGCCCGCAGAGGAAG	XM_015104092.2	262
HAS2	F: GGATCTCCTTCCTCAGCAGTGT R: ATTCCCAGAGGTCCGCTAATG	XM_027560934.1	106
TNFAIP6	F: TGAAAGATGGGATGCATATTGC R: CATTTGGGAAGCCTGGAGATTʹ	NM_001007813.2	101
PTGS2	F: CTTAAACAAGAGCATCCAGAATGG R: GCTGTACGTAGTCTTCAATCACAATCT	NM_174445.2	106
ATPase6	F: GAACACCCACTCCACTAATCCCAAT R: GTGCAAGTGTAGCTCCTCCGATT	MH576694.1	147
ATPase8	F: CACAATCCAGAACTGACACCAACAA R: CGATAAGGGTTACGAGAGGGAGAC	MH576694.1	129
ATP5F1E	F: CAGGCTGGACTCAGCTACATC R: AGTCTTCATGGCGTTTGCTT	XM_027559043.1	96
POLG2	F: CTTCTGGGAAACTACGGGAGAAC R: GTAGCCTCTTGTTTACCAGATCCA	NM_007215.4	84
p21	F: GCAAATATGGGTCTGGGAGA R: AAATAGTCCAGGCCAGGATG	NM_001098958.2	112
mTOR	F: TTAACAGGGTTCGAGAGAAG R: AGAGGTTTTCATGGGATGTC	XM_027564914.1	113
AKT 1	F: AAAAGGAAGTGGTGTACAGG R: GAAGTCGGTGATCTTGATGT	NM_173986.2	80
AKT 2	F: CGACTATCTCAAACTCCTGG R: ATCTTCATGGCATAGTAGCG	NM_001206146.2	90
AKT 3	F: AGCTGTTTTTCCATTTGTCG R: TGTAGATAGTCCAAGGCAGA	NM_001191309.1	94
GADPH	F: CCCAGAATATCATCCCTGCT R: CTGCTTCACCACCTTCTTGA	NM_001034034	185
